# Development of modelling method selection tool for health services management: From problem structuring methods to modelling and simulation methods

**DOI:** 10.1186/1472-6963-11-108

**Published:** 2011-05-19

**Authors:** Gyuchan T Jun, Zoe Morris, Tillal Eldabi, Paul Harper, Aisha Naseer, Brijesh Patel, John P Clarkson

**Affiliations:** 1 Loughborough Design School, Loughborough University, Loughborough, LE11 3TU, UK; 2 Engineering Department, University of Cambridge, Trumpington Street, Cambridge CB2 1PZ, UK; 3 Business School, Brunel University, Uxbridge, Middlesex UB8 3PH, UK; 4 School of Mathematics, Cardiff University, Senghennydd Road, Cardiff CF24 4AG, UK; 5 School of Management, University of Southampton, Southampton SO17 1BJ, UK

## Abstract

**Background:**

There is an increasing recognition that modelling and simulation can assist in the process of designing health care policies, strategies and operations. However, the current use is limited and answers to questions such as *what *methods to use and *when *remain somewhat underdeveloped.

**Aim:**

The aim of this study is to provide a mechanism for decision makers in health services planning and management to compare a broad range of modelling and simulation methods so that they can better select and use them or better commission relevant modelling and simulation work.

**Methods:**

This paper proposes a modelling and simulation method comparison and selection tool developed from a comprehensive literature review, the research team's extensive expertise and inputs from potential users. Twenty-eight different methods were identified, characterised by their relevance to different *application areas, project life cycle stages, types of output *and *levels of insight*, and four input resources required (*time, money, knowledge and data*).

**Results:**

The characterisation is presented in matrix forms to allow quick comparison and selection. This paper also highlights significant knowledge gaps in the existing literature when assessing the applicability of particular approaches to health services management, where modelling and simulation skills are scarce let alone money and time.

**Conclusions:**

A modelling and simulation method comparison and selection tool is developed to assist with the selection of methods appropriate to supporting specific decision making processes. In particular it addresses the issue of which method is most appropriate to which specific health services management problem, what the user might expect to be obtained from the method, and what is required to use the method. In summary, we believe the tool adds value to the scarce existing literature on methods comparison and selection.

## Background

There is an increasing recognition that modelling and simulation can assist in the process of redesigning health services to reconcile expanding demands for health care with cost-containment [1-10]. Policymakers are also keen to capture the benefits of modelling and simulation to healthcare managers [1,2,11-16]. In the English National Health Service (NHS), for example, Primary Care Trusts (PCTs) which purchase health care on behalf of their citizens face a mandatory requirement to undertake some "predictive modelling" to ensure they factor health need into their decisions [10]. Various tools are being developed to assist healthcare managers to model need [17,18]. One example is the *Scenario Generator*, designed to help PCT managers assess the needs of their population and plan care accordingly [19]. The tool allows commissioners to make changes to service provision "virtually" and assess the impact on costs, waits, etc.

Whilst it is not possible to fully gauge the use of modelling and simulation in health services management, it is apparent to expert modellers that the use of modelling and simulation is currently not widespread in the UK [20]. The lack of desire or skill of health care managers to use modelling and simulation, particularly mathematical methods has been highlighted as one of the practical challenges [3,7,11,12,21]. In addition, a need for better awareness and use of a broader set of methods has been recognised to deal with complex and very often political healthcare services management [22-25]. The most appropriate method may be that which fits the problem and the experience of the client and the circumstances in which the modelling and simulation takes place [11-13].

There is value therefore in finding a means to assist healthcare managers to know what methods are available, how they are different, what methods to use and when. The aim of this study is to propose a tool for decision makers in health services planning and management to compare a broad range of modelling and simulation methods so that they can better select and use them or better commission relevant modelling and simulation work. This paper reports on the tool development process and results, specific outcomes are published in a form of a workbook [26] as a part of the results of the RIGHT project (Research Into Global Healthcare Tools); a team of researchers of five UK universities investigated the use of modelling and simulation in health care with a grant from the UK Engineering and Physical Sciences Research Council (EPSRC).

The next section presents the methodology followed for developing the tool and its steps. This is followed by detailed description of results and the paper ends with a discussion and conclusions including lessons learned and future research plans.

## Methods

**Figure 1:**
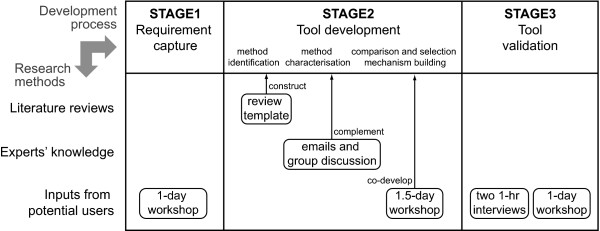
**Research methods used for the tool development process**.

Figure 1 shows three levels of the tool development process and research methods used in each stage. The tool development was primarily based on an extensive review of the literature on the application of modelling and simulation to health care, as well as manufacturing, aerospace and military. The method-related information unobtainable from the literature review was complemented by the research team's extensive expertise in modelling and simulation. Inputs from potential users (health care managers and modelling practitioners) were obtained to capture requirements for the tool, and co-develop and validate it mainly through workshops.

This section explains in further details the three phases of the research process: a comprehensive literature review; use of expert knowledge; and inputs from potential users.

### Literature review

The aim of undertaking the literature review in the RIGHT project was twofold. First, it provided an up-to-date review of simulation and modelling application in health care and other industries. Second, it fed information on the methods (what, where and how they are used) into the comparison and selection tool development. This paper is intended to show the literature review from the perspective of the tool development. The literature review methodology is briefly covered in this paper but more detailed methodology and findings can be found in separate papers [20,27,28].

In summary, the following four topics were searched to find relevant papers using refined search criteria for inclusion and exclusion:

Topic 1. Simulation and modelling in health care;

Topic 2. Simulation and modelling in manufacturing industry;

Topic 3. Simulation and modelling in aerospace and military;

Topic 4. Management and planning methods in health care.

In order to provide a base for tool development, each paper was summarised using a review template which consists of the three categories and sub-fields: method (name, purposes, strength and limitations); problem (specific issue, type, functional area, layer, scenario and setting); resources (time, data, people, expertise, others).

### Experts' knowledge

Despite the comprehensive literature reviews carried out in the study, articles did not always provide all the information required to develop the tool, especially regarding what is required to use the methods in terms of resources such as *time, money, data *and *knowledge*. Expertise of the research team was utilised to complement the information elicited from the literature. The experts' knowledge was continuously captured according to their specialties and cross-checked through communication and group discussions throughout the tool development. The research team consisted of nine academics and seven researchers from five UK universities in the field of knowledge management, operational research & management science, information & communication engineering and systems engineering. Methods each of the research team members particularly specialise in include, but are not limited to, soft OR approaches, conceptual systems modelling, mathematical modelling and simulation techniques. They have extensively applied these methods for planning and management of health care and their experience ranges from 5 years to 30 years.

### Inputs from potential users

Three user engagement workshops in combination with in-depth interviews were carried out to get inputs from potential users. They were by no means a formal test evaluating the effectiveness of the tool, but were meant to provide valuable insights into users' requirements for the tool.

At the early stage of the project, the potential users' requirements for the tool were captured in a one-day workshop, where a dozen health care managers attended. The research team introduced the research objectives and project plans. Then, the health care managers shared their previous model**l**ing experience and expectation for the project through group discussion.

In the middle of the project, i.e. after developing a prototype tool, the research team organized another one and a half day workshop. Sixteen delegates (nine national or local-level health care managers and seven professional modellers) attended and were asked to first review and then redesign the prototype tool. Various suggestions were made from specific wording changes to new overall comparison and selection mechanism. The prototype tool was, then, revised to reflect some of the suggestions from the workshop and printed in the form of a workbook. The workbook was redistributed by post to the health care managers for another review. Then, two one-hour following-up interviews were carried out with local and national level health care managers to investigate their responses in depth.

At the end of the project (after iterative revision of the tool), the national level health care manager at the NHS Institute of Innovation and Improvement (UK' national institute for supporting the National Health Service transformation by developing and spreading new ways of working) wanted to use the workbook [26] for its workshop, 'Building capability in modelling and simulation for commissioning and strategic planning.' The purpose of the workshop was to increase awareness of various modelling and simulation methods and have discussion on how to build capability in these methods. Approximately sixty health care managers (mostly commissioners) attended a full one-day workshop. The tool in the form of the workbook was distributed to each delegate and used as a reference point for group discussion. One of the project team researchers (G. Jun) helped lead group discussion and captured the comments on the utility and usability of the tool throughout the workshop (mostly group feedback session).

The potential users' inputs at these various occasions have been reflected in the tool presented in this paper. Besides, their inputs for the future development of the tool are summarized in the results section. Twenty-eight methods, ranging from problem structuring methods, conceptual modelling methods, mathematical modelling methods to simulation methods, were identified from a commonly applied method list in the literature and through the iterative discussions between the research team members.

## Results

### Method Identification

Table 1 lists simulation and modelling methods applied to different industries in order of popularity: health care; manufacturing; aerospace and military. The general management and planning methods used in health care are listed at the bottom of Table 1, with no particular order. Although the types of methods and the order of popularity were different for each industry to meet their specific needs, commonly applied methods are identified such as Discrete Event Simulation, System Dynamics, Monte Carlo Simulation and Agent-Based Simulation.

**Table 1:** Methods identified for each topic

Topics	Primary methods identified
1. Simulation and modelling in health care	Regression Analysis, Discrete Event Simulation, Mathematical Programming/Optimisation Methods, Markov Models, Queuing Theory, Structural Equation Modelling, System Dynamics, Process Mapping, Spatial Mapping, Monte Carlo Simulation, Cognitive Mapping, Soft Systems Methodology

2. Simulation and modelling in aerospace and the military	Distributed Simulation, Discrete Event Simulation, System Dynamics, Real Time Simulation, Monte Carlo Simulation, Agent Based Simulation, War Gaming, Hybrid Simulation, Inverse Simulation, Petri-net, Markovian Model, Stochastic Combat Simulation

3. Simulation and modelling in manufacturing	Discrete Event Simulation, System Dynamics, Agent-Based Simulation, Monte-Carlo Simulation, Petri-nets, Simulation Gaming, Virtual Simulation, Distributed Simulation

4. Management and planning methods in health care	Lean, Six sigma, Rapid-cycle improvement, Theory of Constraints, Benchmarking, Focus group, Interviews, Narrative approach, Observation, Process analysis, Questionnaire survey, Cognitive task analysis, Action research, Risk analysis

In addition to these commonly applied methods, the research team with expertise in different methods agreed to include additional methods. Qualitative modelling approaches such as various problem structuring methods [29] and conceptual modelling methods [30] were especially expanded based on the expertise of the research team since these types of methods had not been extensively searched in the literature review. It was agreed to identify a broad range of indicative modelling and simulation methods in this project, rather than a full list of comprehensive methods. The method list defined in this project was also agreed to remain open to the possibility of adding or removing at the later stage.

In the end, twenty eight methods were agreed and categorised into four different groups as shown Table 2: five problem structuring methods; eight conceptual modelling methods; seven mathematical modelling methods; eight simulation methods.

**Table 2:** Twenty eight methods identified for the selection tool

Categories	**No**.	Methods
Problem Structuring Methods	1	Drama Theory & Confrontation Analysis
	2	Robustness Analysis
	3	Soft Systems Methodology
	4	Strategic Choice Approach
	5	Strategic Options Development and Analysis

Conceptual Modelling Methods	6	Activity Diagrams
	7	Communication Diagrams
	8	Data Flow Diagrams
	9	Influence Diagrams
	10	Information Diagrams
	11	Issue Maps
	12	State Transition Diagrams
	13	Swim Lane Activity Diagrams

Mathematical Modelling Methods	14	Decision Trees
	15	Markov Modelling
	16	Multivariate Analysis
	17	Optimisation Methods
	18	Petri Nets
	19	Queuing Theory
	20	Survival Analysis

Simulation Methods	21	Agent Based Simulation
	22	Discrete Event Simulation
	23	Gaming Simulation
	24	Hybrid Simulation
	25	Inverse Simulation
	26	Monte Carlo Simulation
	27	Real Time Simulation
	28	System Dynamics

### Method Characterisation by Application Area and Project Life Cycle Stage

First, the list of the eight application areas, drawn from MeSH Terms (Medical Subject Headings), was used for characterising the twenty eight methods (the first column of Table 3). This list was considered most suitable since it covers a broad range of application area without too much overlapping and presumably uses terminology familiar to health care professionals. The lists from the different review topics were found less suitable owing to the industry-specific nature of them e.g. aerospace, military and manufacturing.

**Table 3:** Method characterisation categories by application areas and project lifecycle stages

	Application areas	Project lifecycle stages
1	Policy and strategy planning	Identifying consumer needs for health services

2	Quality management	Developing a new service to meet those needs

3	Risk management	Forecasting the demand for services

4	Financial management	Allocating resources for delivering services

5	Facility planning	Developing plans that will use these resources in delivering services

6	Personnel management	Developing criteria for delivery performance

7	Technology management	Managing the performance of delivery

8	Information/material management	Evaluating the results of health care delivery

Second, the *eight project life cycle stages*, which were drawn from Royston [31], were used for characterising the twenty eight methods (the second column of Table 3).

**Figure 2:**
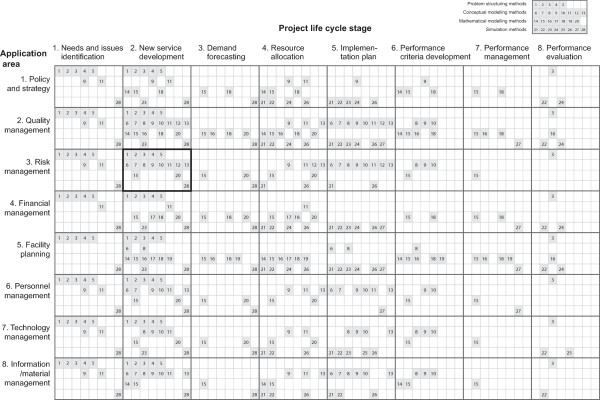
**Method characterisation matrix by application area and project life cycle stage**.

The matches between the twenty eight methods and application area/project life cycle stage were initially made based on the literature and additionally complemented by the experts' knowledge of the research team. Figure 2 shows the matches of which methods are suitable for different combinations of *project life cycle stage *and *application area *using 8 × 8 matrix. Each cell in this matrix consists of a smaller matrix (4 × 8) to show suitable methods. The four rows of the 4 × 8 matrix correspond to the four different groups of methods: the first row to the five problem structuring methods (1~5); the second row to the eight different conceptual modelling methods (6~13); the third row to the seven mathematical modelling methods (14~20); the fourth row to the eight simulation methods (21~28). For example, a problem is about managing risk by identifying and analysing potential hazards and adverse occurrences (third row: *3. risk management*) and at a project life cycle stage of planning new service development (second column: *2. new service development*). Then, the thick black line box in Figure 2 shows that twenty methods are potentially suitable: five problem structuring methods (1~5); eight conceptual modelling methods (6~13); six mathematical modelling methods (14, 15, 16, 18 and 20); two simulation methods (23 and 28).

### Methods Characterisation by Level of Insight and Type of Output

**Figure 3:**
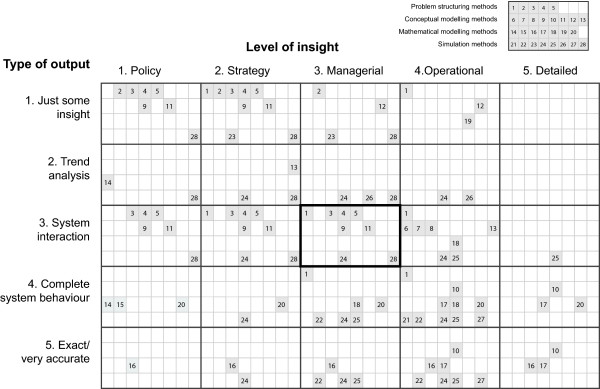
**Method characterisation matrix by type of output and level of insight**.

The twenty eight methods were also characterised in terms of two different output parameters, *level of insight *and *type of output*. The definitions of the five attributes for each output parameter are summarised in Appendix 1. Figure 3 shows the matches of which methods are suitable for different combination of *level of insight *and *type of output *using 5 × 5 matrix in the same way with Figure 2. For example, you expect outputs at managerial *level of insight *(third column: 3. managerial) and want a relatively well-characterised view of the system and how it interacts with the rest of the health care system (third column: 3. system interaction). Then the thick black line box in Figure 3 shows that eight methods are potentially suitable: four problem structuring methods (1, 3, 4 and 5); two conceptual modelling methods (9 and 11); two simulation methods (24 and 28).

### Method Characterisation by Four Input Resources

The twenty eight methods were characterised by four different input resource parameters such as *time, money, knowledge and data*. A five scale index was used to show the ranges of the requirements for each parameter and help users promptly compare them between different methods. Table 4 shows the five scale indices for each parameter, which were determined through internal discussion between the research team and consultation with health care professionals [32]. Our intention was to determine input requirement ranges for conventional application rather than quick-and-dirty application.

**Table 4:** Method characterisation categories by input resource requirements

	Five scale					
		
Input parameters		1	2	3	4	5
Time		hours (t ≤ a day)	days (a day < t ≤ a week)	weeks (a week < t ≤ a month)	months (a month < t ≤ a year)	years (t > a year)

Money		£tens (m ≤ £100)	£hundreds (£100 < m ≤ £1 k)	£thousands (£1 k <m ≤ £10 k)	£10 thousands (£10 k < m≤£100 k)	£100 thousands (m > £100 k)

Knowledge		None	Limited	Moderate	Expert	Complete

Data		None	Guesstimate	Some raw	Good statistics	All types

*Time *is the amount of time required with expertise available, whereas *money *is the amount of money required to purchase hardware, software and expertise. *Knowledge *is not knowledge about specific methods, but qualitative knowledge about problems. *Data *refers to quantitative data required. The definitions of the former two input parameters (*time and money*) are straightforward as shown in Table 4, but the definitions of the latter two input parameters (*knowledge and data*) are summarised in detail in Appendix 2.

Table 5 shows the ranges of the requirements for each method and identifies constraints on the use of candidate methods. For example, one of the simulation methods, 28. System Dynamics is indexed to require the following ranges of the resources: from as short as days to more than a year; from as small as £10s to £10,000s; from moderate to complete knowledge about problems; from no quantitative data to good statistics.

**Table 5:** Method characterisation by the range the input resources required

	Time	Money	Knowledge	Data
Problem Structuring				
1. Drama theory & confrontation analysis				
2. Robustness analysis				
3. Soft systems methodology				
4. Strategic choice approach				
5. Strategic Options Development and Analysis				

Conceptual Modelling				
6. Activity diagrams				
7. Communication diagrams				
8. Data flow diagrams				
9. Influence diagrams				
10. Information diagrams				
11. Issue maps				
12. State transition diagrams				
13. Swim lane activity diagrams				

Mathematical Modelling				
14. Decision trees				
15. Markov modelling				
16. Multivariate analysis				
17. Optimisation methods				
18. Petri nets				
19. Queuing theory				
20. Survival analysis				

Simulation				
21. Agent based simulation				
22. Discrete event simulation				
23. Gaming simulation				
24. Hybrid simulation				
25. Inverse simulation				
26. Monte Carlo simulation				
27. Real-time simulation				
28. System dynamics				

### Method Comparison and Selection Mechanism Building

**Figure 4:**
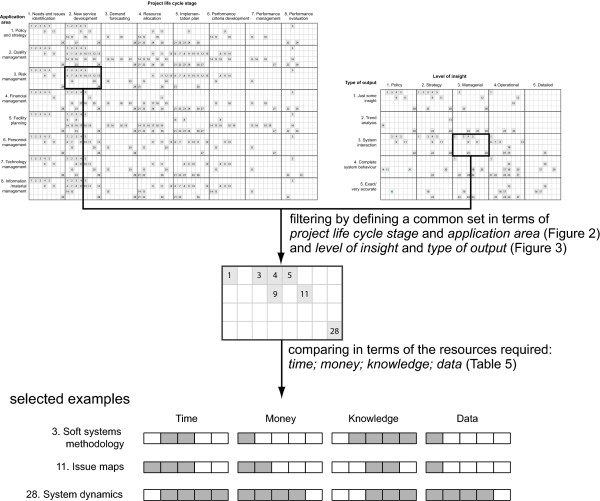
**Method selection tool**.

Figure 4 shows a two-stage method comparison and selection mechanism using two matrices (Figures 2 and 3) and one table (Table 5). The first stage is to filter potential methods using two matrices (Figures 2 and 3) and the second stage is to compare the filtered methods in terms of the four resource requirements (*time, money, knowledge and data*).

The tool is designed to assist selection and comparison of methods appropriate to supporting particular problem situation. For example, a team of health care professionals face the challenge of identifying potential hazards and adverse occurrences (application area: risk management) when planning a new service development (project life cycle stage: new service development). They want to understand how their new services would interact with the rest of the services (type of output: system interaction) at a managerial level (level of insight: managerial). Then, the corresponding common set from two matrices (Figures 2 and 3) shows seven potential methods (1, 3, 4, 5, 9, 11 and 28) as in Figure 4.

The team has not much quantitative data, but they think they know the problems relatively well. Taking into account these constraints, *influence diagrams *or *issue maps *can be applied with moderate investment of time (hours~months) and money (£tens~hundreds). If data, time and money more available, *system dynamics *can be applied, which can additionally support trend analysis. This comparison and selection process enables the selection of methods most suited to the needs and constraints of the particular decision process.

### Tool Validation

Many of the inputs from the co-development workshop were reflected in the current tool, but in-depth interviews and additional workshop provided insight into what is this tool for and how to improve it. What echoed around between the potential users was that the tool was very informative rather than prescriptive. The tool that put a broad range of modelling and simulation methods together with a consistent structure was considered very instrumental in increasing awareness of various methods and their differences. However, many considered that the tool needs to be further improved to convince them to use for the selection of methods. The comments on the further tool development are summarised into the following three main aspects.

First, it was observed that the tool, although originally intended to help identify appropriate methods to problem solving, needs to aid more in problem structuring. A more systematic and phased approach was suggested to understand/formulate problems first before deciding whether or which modelling and simulation may apply. To do that, more exhaustive questions on the symptoms of the problems or the use of the problem structuring methods were proposed to be applied as a part of the tool.

Second, additional information on each method was suggested crucial to the intended use of the tool. In the workbook presentation of the tool [26], each method was additionally described briefly in a page per method regarding its typical functions, purposes and example *application areas*. However, more specific case examples describing how and where each method was applied were considered essential to convince users to push forward with selected methods. In addition, information proving the reliability/authenticity of selected methods such as reference to literature or experts was considered important. Information on practical supports for method application was also suggested needed such as modelling tools and modelling expertise in commercial and academic communities.

Third, there were divergent opinions about the scope of methods. One group of the participants suggested that the current aim for a generic tool across health care may be overambitious. They suggested that the tool be more specific to certain problems and target users, e.g. commissioning, waiting-time target. On the other hand, the other group suggested including general change management methods as well so that they can have a better understanding how the modelling and simulation methods fit with their existing management tools.

## Discussion

### Contributions of the research

We are still far from a definitive tool, if such a tool is indeed possible, but believe that the tool makes a contribution in two major ways. The first is to fill a research practice gap in evidence-based health care management [22] by providing a practical support for the method comparison and selection. Not only can the tool help health care professionals commission more appropriate modelling work, but may also assist health care modelling consultants and researchers to expand their modelling repertoire in order to meet the diverse needs of their health care clients. Research shows that modelling practitioners and researchers tend to select their approaches based on previous experience and competences, despite awareness of other methods [33-35]. They also tend to select from within either a 'hard (quantitative)' or 'soft (qualitative)' paradigm, when they use a number of approaches [36]. Whether the tool helps health care managers find better methods to aid decision-making remains to be seen, but the early feedback from the potential users has been positive and gave valuable insight into the further development.

The second contribution is that the development of the tool has also highlighted significant gaps in knowledge which could be usefully filled. Building the tool from the extant literature proved challenging because studies were often vague when reporting their modelling and simulation process, meaning that information on input resource requirements, such as time, money and data, was often missing. These are important gaps in knowledge when assessing the applicability of particular approaches to health care, where modelling and simulation skills are scarce let alone money and time.

### Extensions to the research

In spite of many debates and discussions throughout the tool development process, we identified the following two research areas to be further addressed.

First, during the development of the tool it became clear that more efforts need to be made in defining the "problem space." Figure 2 shows that the application area does not effectively differentiate the methods, whereas the project life cycle stage better differentiates the methods. It means that the application area used in the tool clearly do not capture the complexity and variety faced by health care managers from the perspective of modelling and simulation application, and nor is there an existing literature which provides adequate insight. Identifying effective parameters and developing meaningful categories requires further characterisation and differentiation by different aspects of problem types. Taxonomy of more specific problem types was suggested to be further developed from the current application area categories and added to the future tool so that candidate methods can be mapped to more specific problem situations. It can also help specify the input resources required and the outputs expected for each method in specific problem situation.

Second, whether and how a comparison and selection tool of this type will actually encourage them increase the range of methods they apply to a problem is an empirical question which we hope to further investigate in time. In the same context, the interface design of this tool, i.e. how different interfaces interact with users during the method comparison and selection, remains an important question that need to be further addressed. In general, health care managers we engaged showed more interest in informative interactions rather than definitive. For example, the two matrices could be informative since they allow users to effectively explore not only methods filtered from their problem and output definitions, but also alternative methods around their definitions. The provision of such overall information was considered to help users redefine their problem and output and learn about capabilities of different methods. Further research in understanding usability and utility of different information visualisation/presentation is needed for the tool to be genuinely accepted by health care users.

## Conclusions

The modelling and simulation method comparison and selection tool is developed to assist health care professionals to commission more appropriate modelling work and health care modelling practitioners or researchers to broaden their selection of methods appropriate to supporting specific decision making processes. In particular it addresses the issue of which method is most appropriate to which specific health care management problem, what the user might expect to be obtained from the method, and what is required to use the method. In summary, we believe the tool adds value to the scarce existing literature on methods comparison and selection. However, we also recognise the limitations of the tool, many of which are reflected in the feedback from the potential users: a more structured problem formulation/structuring stage; and more detailed case examples. Further research is proposed to help address these issues by evaluating and refining the tool closely with healthcare professionals.

## Competing interests

The authors declare that they have no competing interests.

## Authors' contributions

All authors jointly developed the tool and were also involved in organising and running the first two workshops. GTJ conducted the follow-up interviews with potential users, and attended and collected potential users' feedback at the third workshop organized by the NHS Institute. ZM particularly contributed to drafting the background section of the manuscript and GTJ led drafting and revising of the overall manuscript. All authors were involved in drafting, revising and approving the final submitted manuscript.

## Appendices

### Appendix 1 Definitions of the five attributes of two output parameters

• Level of insight: what level of insight do you require from the modelling?

1) Policy: decisions made at national or regional level, e.g. design of public health initiatives with long-term impact such as cancer screening programmes

2) Strategy: major decisions with medium-term impact, e.g. permanently closing a hospital ward, buying an MRI scanner or opening a Walk-in Centre

3) Managerial: e.g. determining nursing staff levels across different specialties in a hospital

4) Operational: e.g. deciding how many fracture clinics to run per week, or how many ICU beds to staff

5) Detailed: e.g. nurse rostering or operating theatre list scheduling

• Level of detail: what level of detail do you require from the modelling?

1) Just some insight: I need to be able to link causes and effects in a general way

2) Trend analysis: I would like to do some simple what-if analysis and to predict any adverse outcomes and patient flows

3) System interactions: I want a relatively well-characterised view of my system and how it interacts with the rest of the health care system

4) Complete system behaviour: I need to understand the complete behaviour of my system and make accurate predictions in terms of intended and unintended outcomes

5) Exact/very accurate: I want an accurate real-time representation of my system running to support an operational decision

### Appendix 2 Definitions of five scales of two input parameters

• Knowledge: what knowledge do you or others have of this problem?

1) New problem: I have no prior knowledge of this problem

2) Limited knowledge: I understand some aspects of this problem, but not others

3) Moderate knowledge: I have access to relevant expertise relating to this problem, but my views of the wider implications are not clear

4) Expert knowledge: I have access to expertise regarding this problem

5) Complete knowledge: I have access to a team of experts capable of understanding this problem

• Quantitative data: what data do you have in order to inform this decision-making?

1) None: I do not have any quantitative data

2) Guesstimate: I can guess a number of variables and have a feel for some trends

3) Some raw data: I am an expert in the field and have access to expert views and some relevant statistics

4) Good statistics: I have good statistics on all aspects of this service, including financial and operational histories

5) Access to all types of data: I can furnish any data that is required and have access to all relevant expertise

## Pre-publication history

The pre-publication history for this paper can be accessed here:

http://www.biomedcentral.com/1472-6963/11/108/prepub
